# Supplementary material for: Learning curve and surgical outcome of robotic assisted colorectal surgery with ERAS program
Source: Sci Rep. 2022 Nov 29;12:20566. doi: 10.1038/s41598-022-24665-w (PMC9709162; doi:10.1038/s41598-022-24665-w)
Supplement: Supplementary file 2 — Supplementary Information 2. [file 41598_2022_24665_MOESM2_ESM.zip › RAL Raw data-Ñ[▒K/119/OP N_ñw╝╨░O▒Kñσ.pdf]

W83 / W83

臺中榮民總醫院
手術室護理記錄

[redacted]

基本資料

- 室別： 開刀房-17室，手術科別： CRS，手術類別： 常規，日期： 2021/10/14
- 進入時間： 1235 ； 麻醉開始時間： 1245 ； Time Out時間： 1357 ； 劃刀時間： 1358 ； 手術結束時間： 1900 送出時間： 1930 ；
- 麻醉方式： GE
  診斷：RECTUM Ca
  手術名稱：Robot Assisted Low Anterior Resection
  手術局部用藥：NIL
  術前預防性抗生素：
  - 抗生素名稱一： Veterin 1GM IVD由麻醉護理師給予
  - 時間： 1350
  - 上次簽章: 羅媗鈴

  ，追加預防性抗生素：
  - 抗生素名稱一： Veterin 1GM IVD由麻醉護理師給予
  - 時間： 1750
  - 上次簽章: 劉雅汶
- 手術醫師： 蔣鋒帆，陳瑨夫，陳凱祥，林順鴻
- 麻醉醫師： VS 齊光彪
-
  - 刷手護士A ： 何佳青，時間： 1235，刷手護士B ： 劉佳潔，時間： 1235，接替刷手一： 林湘芸A，時間： 1500，接替刷手二： 劉雅汶B，時間： 1500
  - 流動護士A ： 羅媗鈴，時間： 1235，接替流動一： 劉雅汶，時間： 1630

皮膚狀況

A-V Shunt： 無
術前： 完整
　記錄者： 上次簽章: 羅媗鈴
術後： 完整
　記錄者： 上次簽章: 劉雅汶

手術準備

手術臥式 單 -手術臥式

-
  - 手術臥式： 截石臥式
  - 使用附件： 矽膠枕頭，腳架
  - 皮膚準備： 10%Aq Betadine，Hibiscrub，2% Chlorhexidine
  - 電燒： 使用電燒: 電極片位置： 其它： 迴路電極墊，記錄者： 上次簽章: 羅媗鈴

止血帶T： 無

植入物

- 植入物名稱/號碼/數量/部位：ENDO WRIST STAPLER/45MM*4.3MM/*1/COLON

，記錄者： 上次簽章: 劉雅汶

標本送檢

- 細菌培養 無
- 細胞學 無
- 冰凍切片 無
- 病理標本
  組織名稱一：Rectum tumor
- 截肢 無

標本交付 標本： 1 袋，標本： 0 盒，標本： 0 瓶，細胞學： 0 管，Culture Tube： 0 管，被交付醫師： CR 陳瑨

夫，

- 記錄者： 上次簽章: 劉雅汶
- 時間： 1800

傷口術後數料

傷口數料：

- 請於圖上標示部位：

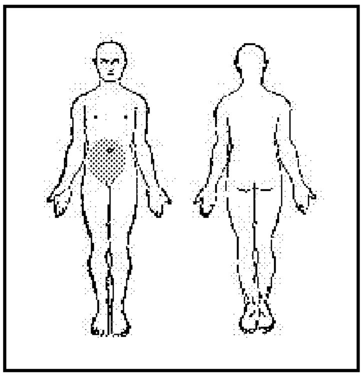

- 傷口數料： mepore

傷口數料：

- 請於圖上標示部位：

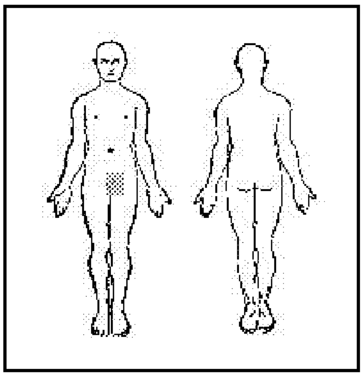

- 傷口數料： PAD

導管

導管	天數	導管資訊	插入日期	插入時間	更換日期	拔除日期	拔除時間	拔除原因	預計拔除/更換	預計拔除/更換日期
Double Lumen CVC(1)	1	號碼:8 Fr; 深度:15cm; 部位:jugular (右); 象限:RU;	2021/10/14	1315						
A-line (1)	1	號碼:20 Ga; 部位:左手;	2021/10/14	1300						
Penrose Drain(1)	1	號碼:12 mm.; 部位:肛門口;	2021/10/14	1840						
Foley(1)	1	號碼:16 Fr.; 部位:膀胱;	2021/10/14	1300						
Epidural PCA(1)	1	部位:脊髓;	2021/10/14	1255						

用物計數

- 手術中出血量： minimal c.c.
- 手術中尿量： 600 c.c.
-
  - 其它： 無

用物計數：

- 部位一： 腹部，1.器械： 正確，2.縫針： 正確，3.刀片： 正確，4.紗布： 正確，5.腹部墊： 正確，6.紗球： 未使用，7.PEANUTS： 未使用，8.紗條： 未使用，9.絲帶： 未使用，10.針頭： 未使用，11.Vessel loop： 未使用，12.Boots： 未使用，13.Jelco： 未使用，14.棉條： 未使用，
  - 第一次：
    - 刷手護士 ： 林湘芸
    - 流動護士： 上次簽章: 劉雅汶
    - 時間： 1715

  ，
  - 第二次：
    - 刷手護士 ： 林湘芸
    - 流動護士： 上次簽章: 劉雅汶
    - 時間： 1720

  ，
  - 第三次：
    - 刷手護士 ： 林湘芸
    - 流動護士： 上次簽章: 劉雅汶
    - 時間： 1730
- 部位二： 肛門，1.器械： 正確，2.縫針： 正確，3.刀片： 正確，4.紗布： 正確，5.腹部墊： 正確，6.紗球： 未使用，7.PEANUTS： 未使用，8.紗條： 未使用，9.絲帶： 未使用，10.針頭： 未使用，11.Vessel loop：

未使用，12.Boots： 未使用，13.Jelco： 未使用，14.棉條： 未使用，

- 第一次：
  - 刷手護士 ： 羅媗鈴
  - 流動護士： 上次簽章: 劉雅汶
  - 時間： 1715

,

- 第二次：
  - 刷手護士 ： 羅媗鈴
  - 流動護士： 上次簽章: 劉雅汶
  - 時間： 1720

,

- 第三次：
  - 刷手護士 ： 羅媗鈴
  - 流動護士： 上次簽章: 劉雅汶
  - 時間： 1730

-
  - 第一次：
    - 流動護士：

,

  - 第二次：
    - 流動護士：

,

  - 第三次：
    - 流動護士：

備註

備註：XI SCOPE 30度 NO.1

- 記錄者： 上次簽章: 羅媗鈴
- 時間： 1340

填寫護理人員 劉雅汶

正式摘要

W83 / W83

doctorRank:B
